# Supplementary figures and images for: Structure of the EphB6 receptor ectodomain
Source: PLoS One. 2021 Mar 26;16(3):e0247335. doi: 10.1371/journal.pone.0247335 (PMC7997048; doi:10.1371/journal.pone.0247335)

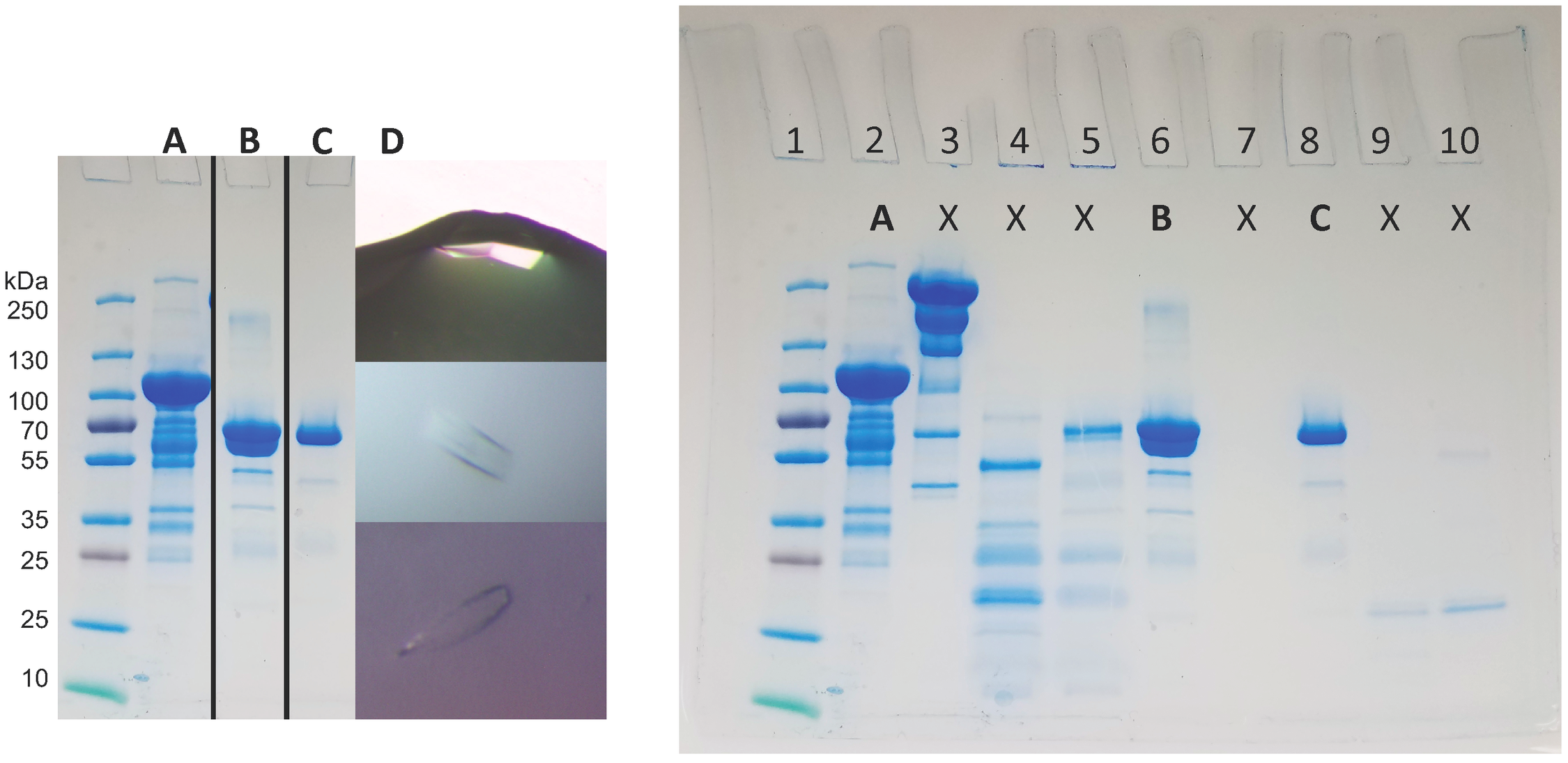

Supplement: S1 Fig — (TIF) [file pone.0247335.s001.tif]
