# Supplementary material for: Structure of the EphB6 receptor ectodomain
Source: PLoS One. 2021 Mar 26;16(3):e0247335. doi: 10.1371/journal.pone.0247335 (PMC7997048; doi:10.1371/journal.pone.0247335)
Supplement: S1 File — (PDF) [file pone.0247335.s002.pdf]

Full wwPDB X-ray Structure Validation Report (i)

Sep 24, 2020 – 04:44 PM EDT

PDB ID	:	7K7J
Title	:	EphB6 receptor ectodomain
Deposited on	:	2020-09-22
Resolution	:	3.00 Å(reported)

This is a Full wwPDB X-ray Structure Validation Report.

This report is produced by the wwPDB biocuration pipeline after annotation of the structure.

We welcome your comments at *validation@mail.wwpdb.org*
A user guide is available at
https://www.wwpdb.org/validation/2017/XrayValidationReportHelp
with specific help available everywhere you see the (i) symbol.

The following versions of software and data (see references (i)) were used in the production of this report:

MolProbity	:	4.02b-467
Mogul	:	1.8.5 (274361), CSD as541be (2020)
Xtriage (Phenix)	:	1.13
EDS	:	2.14.6
Percentile statistics	:	20191225.v01 (using entries in the PDB archive December 25th 2019)
Refmac	:	5.8.0158
CCP4	:	7.0.044 (Gargrove)
Ideal geometry (proteins)	:	Engh & Huber (2001)
Ideal geometry (DNA, RNA)	:	Parkinson et al. (1996)
Validation Pipeline (wwPDB-VP)	:	2.14.6

CONFIDENTIAL VALIDATION REPORT

1 Overall quality at a glance (i)

The following experimental techniques were used to determine the structure:
X-RAY DIFFRACTION

The reported resolution of this entry is 3.00 Å.

Percentile scores (ranging between 0-100) for global validation metrics of the entry are shown in the following graphic. The table shows the number of entries on which the scores are based.

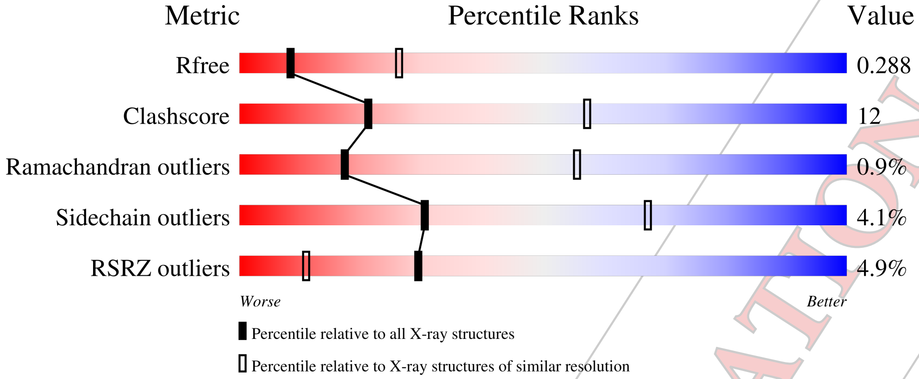

Metric	Whole archive (#Entries)	Similar resolution (#Entries, resolution range(Å))
R_{free}	130704	2092 (3.00-3.00)
Clashscore	141614	2416 (3.00-3.00)
Ramachandran outliers	138981	2333 (3.00-3.00)
Sidechain outliers	138945	2336 (3.00-3.00)
RSRZ outliers	127900	1990 (3.00-3.00)

The table below summarises the geometric issues observed across the polymeric chains and their fit to the electron density. The red, orange, yellow and green segments on the lower bar indicate the fraction of residues that contain outliers for >=3, 2, 1 and 0 types of geometric quality criteria respectively. A grey segment represents the fraction of residues that are not modelled. The numeric value for each fraction is indicated below the corresponding segment, with a dot representing fractions <=5% The upper red bar (where present) indicates the fraction of residues that have poor fit to the electron density. The numeric value is given above the bar.

Mol	Chain	Length	Quality of chain
1	A	547	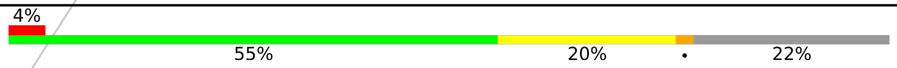
2	B	2	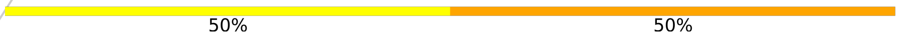

CONFIDENTIAL VALIDATION REPORT

2 Entry composition (i)

There are 2 unique types of molecules in this entry. The entry contains 3255 atoms, of which 0 are hydrogens and 0 are deuteriums.

In the tables below, the ZeroOcc column contains the number of atoms modelled with zero occupancy, the AltConf column contains the number of residues with at least one atom in alternate conformation and the Trace column contains the number of residues modelled with at most 2 atoms.

- Molecule 1 is a protein called Ephrin type-B receptor 6.

Mol	Chain	Residues	Atoms					ZeroOcc	AltConf	Trace
			Total	C	N	O	S			
1	A	429	3227	2001	593	611	22	0	0	0

- Molecule 2 is an oligosaccharide called 2-acetamido-2-deoxy-beta-D-glucopyranose-(1-4)-2-acetamido-2-deoxy-beta-D-glucopyranose.

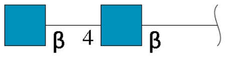

Mol	Chain	Residues	Atoms				ZeroOcc	AltConf	Trace
			Total	C	N	O			
2	B	2	28	16	2	10	0	0	0

CONFIDENTIAL VALIDATION REPORT

3 Residue-property plots (i)

These plots are drawn for all protein, RNA, DNA and oligosaccharide chains in the entry. The first graphic for a chain summarises the proportions of the various outlier classes displayed in the second graphic. The second graphic shows the sequence view annotated by issues in geometry and electron density. Residues are color-coded according to the number of geometric quality criteria for which they contain at least one outlier: green = 0, yellow = 1, orange = 2 and red = 3 or more. A red dot above a residue indicates a poor fit to the electron density (RSRZ > 2). Stretches of 2 or more consecutive residues without any outlier are shown as a green connector. Residues present in the sample, but not in the model, are shown in grey.

• Molecule 1: Ephrin type-B receptor 6

Chain A:

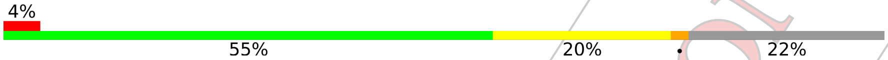

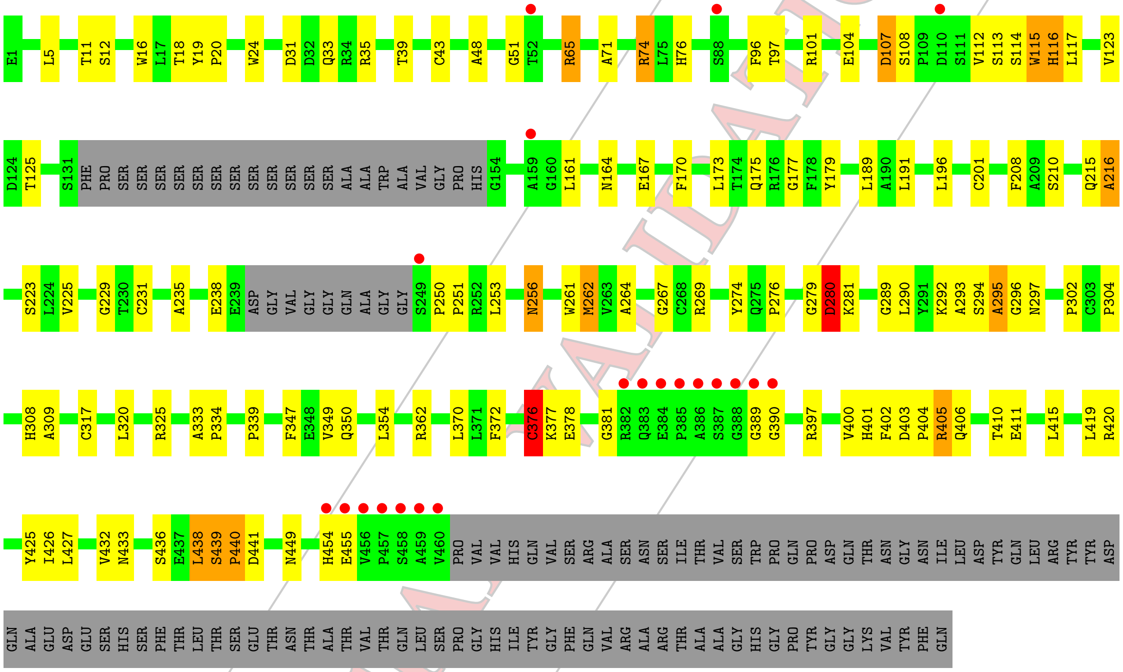

• Molecule 2: 2-acetamido-2-deoxy-beta-D-glucopyranose-(1-4)-2-acetamido-2-deoxy-beta-D-glucopyranose

Chain B:

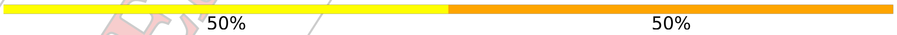

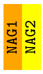

CONFIDENTIAL VALIDATION REPORT

4 Data and refinement statistics (i)

Property	Value	Source
Space group	P 43 21 2	Depositor
Cell constants<br>a, b, c, α, β, γ	105.31Å 105.31Å 188.72Å<br>90.00° 90.00° 90.00°	Depositor
Resolution (Å)	48.05 – 3.00<br>48.06 – 3.00	Depositor<br>EDS
% Data completeness<br>(in resolution range)	99.8 (48.05-3.00)<br>99.9 (48.06-3.00)	Depositor<br>EDS
R_{merge}	(Not available)	Depositor
R_{sym}	(Not available)	Depositor
$< I/\sigma(I) >$ [1]	1.92 (at 3.01Å)	Xtriage
Refinement program	PHENIX (1.16_3549: ???)	Depositor
R, R_{free}	0.235 , 0.288<br>0.234 , 0.288	Depositor<br>DCC
R_{free} test set	1046 reflections (4.78%)	wwPDB-VP
Wilson B-factor (Å^2)	84.1	Xtriage
Anisotropy	0.243	Xtriage
Bulk solvent k_{sol}(e/Å^3), B_{sol}(Å^2)	0.32 , 57.7	EDS
L-test for twinning[2]	$< \|L\| > = 0.49$, $< L^2 > = 0.32$	Xtriage
Estimated twinning fraction	No twinning to report.	Xtriage
F_o,F_c correlation	0.92	EDS
Total number of atoms	3255	wwPDB-VP
Average B, all atoms (Å^2)	90.0	wwPDB-VP

Xtriage's analysis on translational NCS is as follows: *The largest off-origin peak in the Patterson function is 3.02% of the height of the origin peak. No significant pseudotranslation is detected.*

[1] Intensities estimated from amplitudes.
[2] Theoretical values of $< |L| >$, $< L^2 >$ for acentric reflections are 0.5, 0.333 respectively for untwinned datasets, and 0.375, 0.2 for perfectly twinned datasets.

CONFIDENTIAL VALIDATION REPORT

5 Model quality (i)

5.1 Standard geometry (i)

Bond lengths and bond angles in the following residue types are not validated in this section: NAG

The Z score for a bond length (or angle) is the number of standard deviations the observed value is removed from the expected value. A bond length (or angle) with $|Z| > 5$ is considered an outlier worth inspection. RMSZ is the root-mean-square of all Z scores of the bond lengths (or angles).

Mol	Chain	Bond lengths		Bond angles	
		RMSZ	#$|Z|$ >5	RMSZ	#$|Z|$ >5
1	A	0.54	1/3312 (0.0%)	0.78	2/4511 (0.0%)

Chiral center outliers are detected by calculating the chiral volume of a chiral center and verifying if the center is modelled as a planar moiety or with the opposite hand.A planarity outlier is detected by checking planarity of atoms in a peptide group, atoms in a mainchain group or atoms of a sidechain that are expected to be planar.

Mol	Chain	#Chirality outliers	#Planarity outliers
1	A	0	15

All (1) bond length outliers are listed below:

Mol	Chain	Res	Type	Atoms	Z	Observed(Å)	Ideal(Å)
1	A	43	CYS	CB-SG	-5.06	1.73	1.81

All (2) bond angle outliers are listed below:

Mol	Chain	Res	Type	Atoms	Z	Observed(^{o})	Ideal(^{o})
1	A	439	SER	N-CA-CB	7.06	121.09	110.50
1	A	376	CYS	CA-CB-SG	-5.58	103.95	114.00

There are no chirality outliers.

All (15) planarity outliers are listed below:

Mol	Chain	Res	Type	Group
1	A	107	ASP	Peptide
1	A	11	THR	Peptide
1	A	114	SER	Peptide
1	A	215	GLN	Peptide

Continued on next page...

CONFIDENTIAL VALIDATION REPORT

Continued from previous page...

Mol	Chain	Res	Type	Group
1	A	216	ALA	Peptide
1	A	250	PRO	Peptide
1	A	262	MET	Peptide
1	A	293	ALA	Peptide
1	A	295	ALA	Peptide
1	A	33	GLN	Peptide
1	A	436	SER	Peptide
1	A	438	LEU	Peptide
1	A	439	SER	Peptide
1	A	440	PRO	Peptide
1	A	65	ARG	Peptide

5.2 Too-close contacts (i)

In the following table, the Non-H and H(model) columns list the number of non-hydrogen atoms and hydrogen atoms in the chain respectively. The H(added) column lists the number of hydrogen atoms added and optimized by MolProbity. The Clashes column lists the number of clashes within the asymmetric unit, whereas Symm-Clashes lists symmetry related clashes.

Mol	Chain	Non-H	H(model)	H(added)	Clashes	Symm-Clashes
1	A	3227	0	3069	77	0
2	B	28	0	25	2	0
All	All	3255	0	3094	77	0

The all-atom clashscore is defined as the number of clashes found per 1000 atoms (including hydrogen atoms). The all-atom clashscore for this structure is 12.

All (77) close contacts within the same asymmetric unit are listed below, sorted by their clash magnitude.

Atom-1	Atom-2	Interatomic distance (Å)	Clash overlap (Å)
1:A:325:ARG:NH1	1:A:333:ALA:O	2.08	0.87
1:A:115:TRP:O	1:A:117:LEU:N	2.11	0.80
1:A:104:GLU:HG3	1:A:175:GLN:HB3	1.66	0.76
1:A:402:PHE:CD2	1:A:406:GLN:HB2	2.24	0.72
1:A:65:ARG:HB2	1:A:177:GLY:HA2	1.70	0.72
1:A:71:ALA:HB3	1:A:170:PHE:HD2	1.53	0.71
1:A:18:THR:HG21	1:A:24:TRP:H	1.56	0.70
1:A:96:PHE:HB3	1:A:189:LEU:HD11	1.77	0.66
1:A:376:CYS:SG	1:A:377:LYS:N	2.69	0.65
1:A:65:ARG:HD3	1:A:175:GLN:O	1.96	0.65

Continued on next page...

CONFIDENTIAL VALIDATION REPORT

Continued from previous page...

Atom-1	Atom-2	Interatomic distance (Å)	Clash overlap (Å)
1:A:19:TYR:HB3	1:A:20:PRO:HD3	1.79	0.62
1:A:97:THR:HG22	1:A:125:THR:HG23	1.82	0.61
1:A:317:CYS:O	1:A:325:ARG:NH2	2.34	0.60
1:A:304:PRO:HB2	1:A:334:PRO:HA	1.85	0.59
1:A:347:PHE:HD1	1:A:354:LEU:HD11	1.70	0.57
1:A:410:THR:OG1	1:A:411:GLU:OE1	2.21	0.57
1:A:290:LEU:HD22	1:A:302:PRO:HA	1.87	0.56
1:A:208:PHE:HZ	1:A:276:PRO:HB3	1.71	0.56
1:A:292:LYS:NZ	1:A:296:GLY:O	2.28	0.55
1:A:71:ALA:HB3	1:A:170:PHE:CD2	2.38	0.55
1:A:208:PHE:CZ	1:A:276:PRO:HB3	2.43	0.54
1:A:405:ARG:O	1:A:406:GLN:HB3	2.08	0.54
1:A:347:PHE:CD1	1:A:354:LEU:HD11	2.43	0.53
1:A:107:ASP:HB2	1:A:179:TYR:OH	2.09	0.52
1:A:101:ARG:NH2	1:A:107:ASP:OD2	2.43	0.52
1:A:269:ARG:NH1	1:A:295:ALA:HB1	2.24	0.52
1:A:115:TRP:CD1	1:A:115:TRP:N	2.74	0.51
1:A:5:LEU:HB3	1:A:196:LEU:HB2	1.92	0.51
1:A:65:ARG:HD2	1:A:173:LEU:HB3	1.93	0.51
1:A:251:PRO:HB3	1:A:267:GLY:HA2	1.93	0.50
1:A:370:LEU:HD21	1:A:372:PHE:CZ	2.45	0.50
1:A:449:ASN:ND2	2:B:1:NAG:O7	2.45	0.50
1:A:238:GLU:HG2	1:A:267:GLY:O	2.12	0.50
1:A:290:LEU:HD22	1:A:302:PRO:CA	2.42	0.50
1:A:231:CYS:HB3	1:A:235:ALA:HB3	1.94	0.49
1:A:123:VAL:HG21	1:A:170:PHE:HE1	1.77	0.49
1:A:253:LEU:HD13	1:A:264:ALA:HB2	1.94	0.49
1:A:403:ASP:HB2	1:A:415:LEU:HB3	1.95	0.48
1:A:253:LEU:HG	1:A:261:TRP:CE3	2.49	0.48
1:A:370:LEU:HD12	1:A:432:VAL:O	2.14	0.48
1:A:362:ARG:HE	1:A:362:ARG:HB3	1.42	0.47
1:A:39:THR:HB	1:A:191:LEU:O	2.14	0.47
1:A:378:GLU:HG2	1:A:425:TYR:CE2	2.49	0.46
1:A:339:PRO:HD3	1:A:433:ASN:ND2	2.31	0.46
1:A:438:LEU:HD23	1:A:438:LEU:HA	1.50	0.46
1:A:208:PHE:CZ	1:A:280:ASP:HA	2.50	0.46
1:A:18:THR:CG2	1:A:24:TRP:H	2.28	0.46
1:A:377:LYS:HB2	1:A:426:ILE:CG2	2.46	0.45
1:A:16:TRP:N	1:A:16:TRP:CD1	2.84	0.45
1:A:161:LEU:HD12	1:A:161:LEU:HA	1.88	0.45
1:A:269:ARG:HH11	1:A:295:ALA:HB1	1.82	0.45

Continued on next page...

CONFIDENTIAL VALIDATION REPORT

Continued from previous page...

Atom-1	Atom-2	Interatomic distance (Å)	Clash overlap (Å)
1:A:349:VAL:C	1:A:350:GLN:HG2	2.37	0.45
1:A:454:HIS:O	1:A:455:GLU:HG3	2.17	0.45
1:A:389:GLY:HA2	2:B:2:NAG:H81	1.97	0.45
1:A:115:TRP:C	1:A:117:LEU:H	2.12	0.44
1:A:201:CYS:HB3	1:A:261:TRP:CE2	2.52	0.44
1:A:403:ASP:CB	1:A:415:LEU:HB3	2.48	0.43
1:A:216:ALA:O	1:A:225:VAL:HG21	2.17	0.43
1:A:381:GLY:HA2	1:A:390:GLY:O	2.19	0.43
1:A:210:SER:O	1:A:229:GLY:HA2	2.18	0.43
1:A:116:HIS:CG	1:A:116:HIS:O	2.72	0.43
1:A:76:HIS:HA	1:A:164:ASN:O	2.18	0.42
1:A:292:LYS:HE2	1:A:294:SER:O	2.20	0.42
1:A:256:ASN:HB2	1:A:262:MET:CG	2.50	0.41
1:A:48:ALA:O	1:A:51:GLY:N	2.50	0.41
1:A:308:HIS:ND1	1:A:320:LEU:HD23	2.35	0.41
1:A:97:THR:HA	1:A:125:THR:HA	2.02	0.41
1:A:279:GLY:O	1:A:281:LYS:HB2	2.20	0.41
1:A:289:GLY:CA	1:A:309:ALA:HB3	2.51	0.41
1:A:400:VAL:O	1:A:401:HIS:C	2.59	0.41
1:A:123:VAL:HG21	1:A:170:PHE:CE1	2.55	0.41
1:A:274:TYR:O	1:A:292:LYS:HD3	2.21	0.41
1:A:427:LEU:HA	1:A:427:LEU:HD23	1.83	0.40
1:A:108:SER:HB3	1:A:112:VAL:HB	2.03	0.40
1:A:74:ARG:HD3	1:A:167:GLU:OE1	2.21	0.40
1:A:402:PHE:CG	1:A:406:GLN:HB2	2.55	0.40
1:A:440:PRO:HA	1:A:441:ASP:O	2.22	0.40

There are no symmetry-related clashes.

5.3 Torsion angles (i)

5.3.1 Protein backbone (i)

In the following table, the Percentiles column shows the percent Ramachandran outliers of the chain as a percentile score with respect to all X-ray entries followed by that with respect to entries of similar resolution.

The Analysed column shows the number of residues for which the backbone conformation was analysed, and the total number of residues.

CONFIDENTIAL VALIDATION REPORT

Mol	Chain	Analysed	Favoured	Allowed	Outliers	Percentiles	
1	A	423/547 (77%)	349 (82%)	70 (16%)	4 (1%)	17	55

All (4) Ramachandran outliers are listed below:

Mol	Chain	Res	Type
1	A	116	HIS
1	A	280	ASP
1	A	12	SER
1	A	404	PRO

5.3.2 Protein sidechains (i)

In the following table, the Percentiles column shows the percent sidechain outliers of the chain as a percentile score with respect to all X-ray entries followed by that with respect to entries of similar resolution.

The Analysed column shows the number of residues for which the sidechain conformation was analysed, and the total number of residues.

Mol	Chain	Analysed	Rotameric	Outliers	Percentiles	
1	A	339/434 (78%)	325 (96%)	14 (4%)	30	67

All (14) residues with a non-rotameric sidechain are listed below:

Mol	Chain	Res	Type
1	A	31	ASP
1	A	35	ARG
1	A	74	ARG
1	A	113	SER
1	A	115	TRP
1	A	223	SER
1	A	256	ASN
1	A	280	ASP
1	A	297	ASN
1	A	376	CYS
1	A	397	ARG
1	A	405	ARG
1	A	419	LEU
1	A	420	ARG

Some sidechains can be flipped to improve hydrogen bonding and reduce clashes. There are no such sidechains identified.

CONFIDENTIAL VALIDATION REPORT

5.3.3 RNA ⓘ

There are no RNA molecules in this entry.

5.4 Non-standard residues in protein, DNA, RNA chains ⓘ

There are no non-standard protein/DNA/RNA residues in this entry.

5.5 Carbohydrates ⓘ

2 monosaccharides are modelled in this entry.

In the following table, the Counts columns list the number of bonds (or angles) for which Mogul statistics could be retrieved, the number of bonds (or angles) that are observed in the model and the number of bonds (or angles) that are defined in the Chemical Component Dictionary. The Link column lists molecule types, if any, to which the group is linked. The Z score for a bond length (or angle) is the number of standard deviations the observed value is removed from the expected value. A bond length (or angle) with $|Z| > 2$ is considered an outlier worth inspection. RMSZ is the root-mean-square of all Z scores of the bond lengths (or angles).

Mol	Type	Chain	Res	Link	Bond lengths			Bond angles		
					Counts	RMSZ	$\#\|Z\| > 2$	Counts	RMSZ	$\#\|Z\| > 2$
2	NAG	B	1	1,2	14,14,15	0.62	1 (7%)	17,19,21	0.56	0
2	NAG	B	2	2	14,14,15	0.30	0	17,19,21	0.52	0

In the following table, the Chirals column lists the number of chiral outliers, the number of chiral centers analysed, the number of these observed in the model and the number defined in the Chemical Component Dictionary. Similar counts are reported in the Torsion and Rings columns. '-' means no outliers of that kind were identified.

Mol	Type	Chain	Res	Link	Chirals	Torsions	Rings
2	NAG	B	1	1,2	-	3/6/23/26	0/1/1/1
2	NAG	B	2	2	-	1/6/23/26	0/1/1/1

All (1) bond length outliers are listed below:

Mol	Chain	Res	Type	Atoms	Z	Observed(Å)	Ideal(Å)
2	B	1	NAG	C1-C2	2.17	1.55	1.52

There are no bond angle outliers.

There are no chirality outliers.

All (4) torsion outliers are listed below:

CONFIDENTIAL VALIDATION REPORT

Mol	Chain	Res	Type	Atoms
2	B	1	NAG	O5-C5-C6-O6
2	B	1	NAG	C4-C5-C6-O6
2	B	2	NAG	O5-C5-C6-O6
2	B	1	NAG	C3-C2-N2-C7

There are no ring outliers.

2 monomers are involved in 2 short contacts:

Mol	Chain	Res	Type	Clashes	Symm-Clashes
2	B	1	NAG	1	0
2	B	2	NAG	1	0

The following is a two-dimensional graphical depiction of Mogul quality analysis of bond lengths, bond angles, torsion angles, and ring geometry for oligosaccharide.

Oligosaccharide Chain B

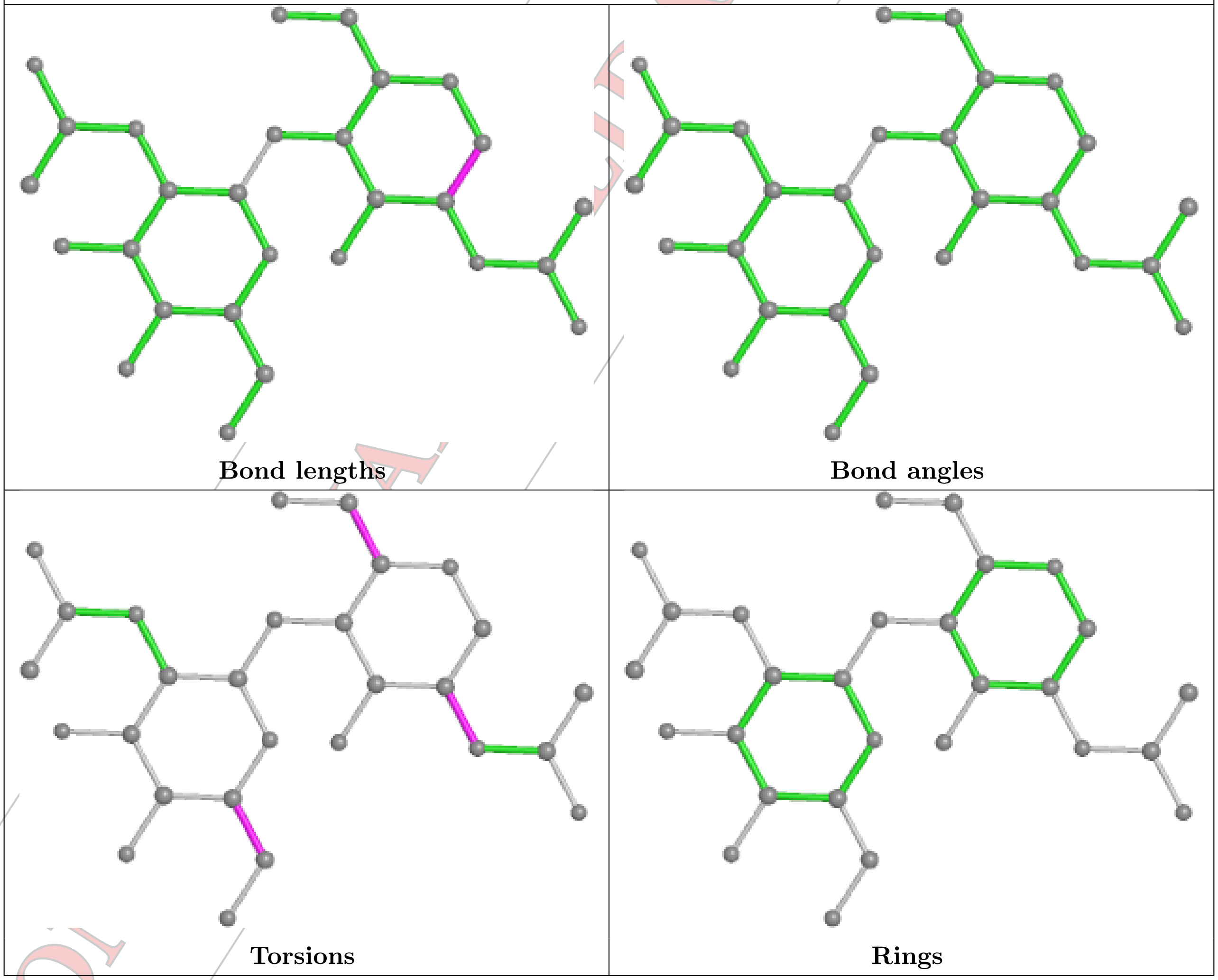

5.6 Ligand geometry (i)

There are no ligands in this entry.

5.7 Other polymers (i)

There are no such residues in this entry.

5.8 Polymer linkage issues (i)

There are no chain breaks in this entry.

CONFIDENTIAL VALIDATION REPORT

6 Fit of model and data (i)

6.1 Protein, DNA and RNA chains (i)

In the following table, the column labelled '#RSRZ> 2' contains the number (and percentage) of RSRZ outliers, followed by percent RSRZ outliers for the chain as percentile scores relative to all X-ray entries and entries of similar resolution. The OWAB column contains the minimum, median, 95th percentile and maximum values of the occupancy-weighted average B-factor per residue. The column labelled 'Q< 0.9' lists the number of (and percentage) of residues with an average occupancy less than 0.9.

Mol	Chain	Analysed	<RSRZ>	#RSRZ>2			OWAB(Å^2)	Q<0.9
1	A	429/547 (78%)	0.12	21 (4%)	29	11	44, 79, 176, 249	0

All (21) RSRZ outliers are listed below:

Mol	Chain	Res	Type	RSRZ
1	A	386	ALA	10.9
1	A	387	SER	8.9
1	A	384	GLU	7.8
1	A	459	ALA	6.3
1	A	383	GLN	5.7
1	A	456	VAL	5.1
1	A	460	VAL	5.1
1	A	385	PRO	5.0
1	A	388	GLY	4.7
1	A	458	SER	4.7
1	A	110	ASP	4.1
1	A	454	HIS	4.0
1	A	382	ARG	3.8
1	A	249	SER	3.7
1	A	457	PRO	3.6
1	A	389	GLY	2.8
1	A	390	GLY	2.3
1	A	455	GLU	2.3
1	A	52	THR	2.3
1	A	88	SER	2.3
1	A	159	ALA	2.1

6.2 Non-standard residues in protein, DNA, RNA chains (i)

There are no non-standard protein/DNA/RNA residues in this entry.

CONFIDENTIAL VALIDATION REPORT

6.3 Carbohydrates (i)

In the following table, the Atoms column lists the number of modelled atoms in the group and the number defined in the chemical component dictionary. The B-factors column lists the minimum, median, 95th percentile and maximum values of B factors of atoms in the group. The column labelled 'Q< 0.9' lists the number of atoms with occupancy less than 0.9.

Mol	Type	Chain	Res	Atoms	RSCC	RSR	B-factors(Å^2)	Q<0.9
2	NAG	B	2	14/15	0.77	0.27	165,190,194,197	0
2	NAG	B	1	14/15	0.88	0.16	124,147,183,183	0

The following is a graphical depiction of the model fit to experimental electron density for oligosaccharide. Each fit is shown from different orientation to approximate a three-dimensional view.

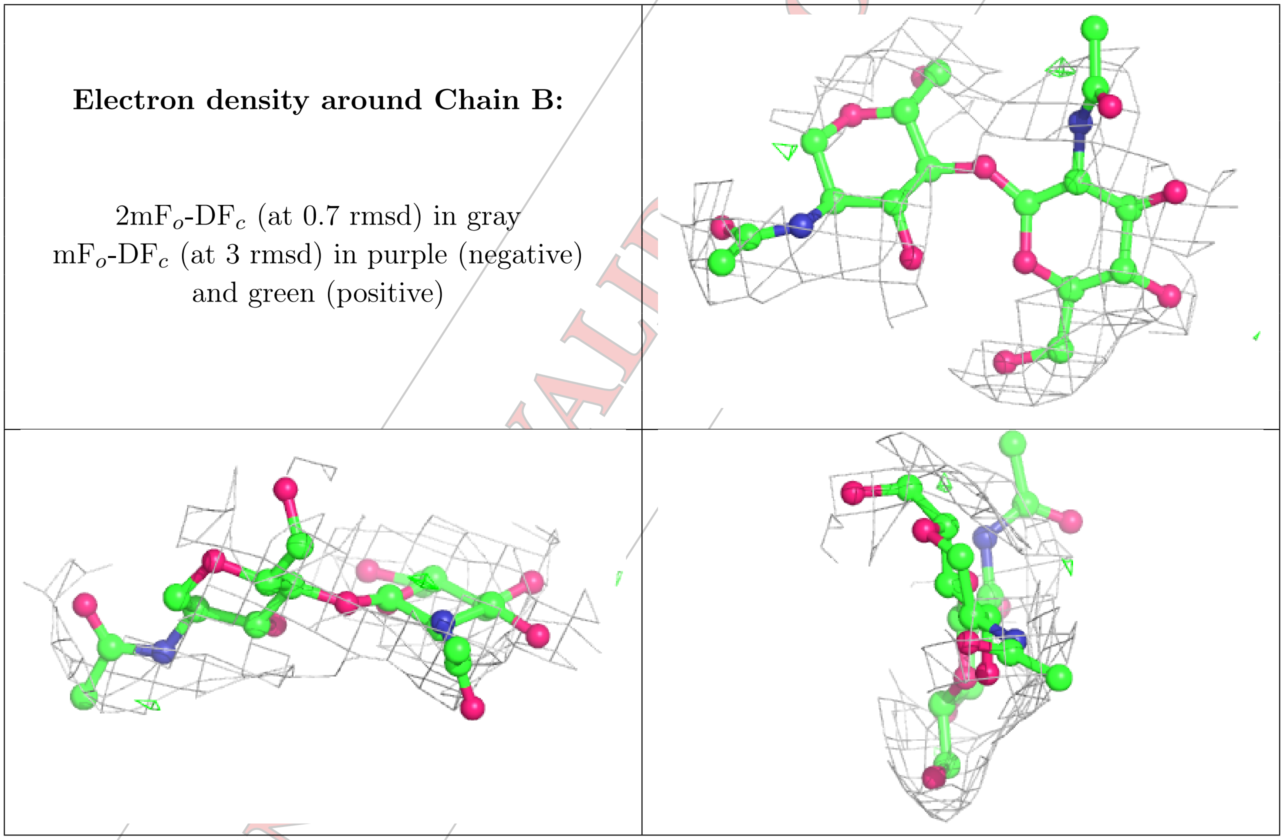

6.4 Ligands (i)

There are no ligands in this entry.

6.5 Other polymers (i)

There are no such residues in this entry.

CONFIDENTIAL VALIDATION REPORT
