# Supplementary material for: Structure of the EphB6 receptor ectodomain
Source: PLoS One. 2021 Mar 26;16(3):e0247335. doi: 10.1371/journal.pone.0247335 (PMC7997048; doi:10.1371/journal.pone.0247335)
Supplement: Attachment — Submitted filename: Response_to_Reviewers.docx [file pone.0247335.s003.docx]

New York, 27 January 2021

Dear PlosOne Editors,

Enclosed, please find our revised manuscript entitled “Structure of the EphB6 receptor ectodomain” that we are submitting for publication in PlosOne. We would like to thank the reviewers for their thoughtful comments and suggestions. Indeed, we believe these suggestions have made the manuscript stronger. We were happy to read that the reviewers find the work “well documented, well-written, and informative”. We made every effort to address the specific suggestions of the reviewers to modify Figure 5 and to comment on the internalization of EphB6 receptor upon the interaction with agonistic agents/ligands. We hope these changes are satisfactory to allow the publication of our manuscript.

All revisions that we have made to the text are listed below:

1) We have added an arrow showing the position of Ser-148 in Figure 5. This is the residue preceding the ‘Super-Serine’ loop. Since the loop is not structured in our model, this is, we feel, the best way to indicate that the loop, indeed, is part of the initial ligand-binding module, while the loop 125-130 is located on the opposite side of the EphB6-LBD, on the surface area that participates in the formation of the Eph/ephrin heterotetramers.
2) We have modified the Figure 5 legend to indicate the change we made to this figure.
3) We have added references and sentences at the end of the Results section to emphasize that both EphB6 and EphA2 get internalized upon ligand/agonist interaction, though possibly with different dynamics.
4) We have also added a sentence on the possibility that the changes in the internalization dynamics might be the result of the different sizes of the head-to-tail interaction surface areas. While we are currently experimenting further on these homotypic interactions, a precise quantification of the interactions needs a new comprehensive study.

Please let us know if there is any other information or materials you would need.

Sincerely yours,

Juha-P. Himanen

Sloan-Kettering Institute

New York

[himanenj@mskcc.org](mailto:himanenj@mskcc.org)
